# Efficacy and safety of triple therapy containing berberine hydrochloride, amoxicillin, and rabeprazole in the eradication of *Helicobacter pylori*

**DOI:** 10.1111/1751-2980.13146

**Published:** 2022-12-21

**Authors:** Xing Xing Chen, Yu Xin Chen, Han Xin Bi, Xin Zhao, Li Feng Zhang, Jun Ye Liu, Yong Quan Shi

**Affiliations:** ^1^ Xi'an Medical University Xi'an Shaanxi Province China; ^2^ State Key Laboratory of Cancer Biology, National Clinical Research Center for Digestive Diseases and Xijing Hospital Air Force Medical University Xi'an Shaanxi Province China; ^3^ The Second Affiliated Hospital of Xi'an Medical College Xi'an Shaanxi Province China; ^4^ Department of Radiation Protective Medicine Air Force Medical University Xi'an Shaanxi Province China

**Keywords:** berberine, eradication, *Helicobacter pylori*, quadruple therapy

## Abstract

**Objective:**

To estimate the effectiveness and safety of triple therapy containing berberine, amoxicillin, and rabeprazole in the eradication of *Helicobacter pylori* (*H. pylori*)*.*

**Methods:**

This prospective, randomized controlled, open‐label, noninferiority trial included treatment‐naive patients with *H. pylori* infection who were randomly allocated at a ratio of 1:1 into the berberine triple therapy group (berberine hydrochloride 300 mg thrice daily, amoxicillin 1 g twice daily, and rabeprazole 10 mg twice daily) or standard bismuth‐containing quadruple therapy group (amoxicillin 1 g twice daily, rabeprazole 10 mg twice daily, clarithromycin 500 mg twice daily, and bismuth tartrate 200 mg twice daily) for 14 days. Negative ^13^C/^14^C‐urea breath test at 4 weeks after completion of the therapy was regarded as successful eradication.

**Results:**

Altogether 262 and 262 patients received berberine triple therapy and bismuth‐containing quadruple therapy, respectively. Both intention‐to‐treat (79.8% vs 80.9%, *P* = 0.742) and per‐protocol analyses (83.6% and 85.1%, *P* = 0.636) showed comparable eradication rate between the two groups, indicating a noninferior eradication rate (the lower limit of the 95% confidence interval over −10% [−7.9% and −7.87%, respectively]). Adverse events more commonly occurred in the bismuth‐containing quadruple‐therapy group (8.8% vs 16.0%, *P* = 0.012), while patient compliance and symptom improvement of the two regimens were comparable.

**Conclusion:**

Triple therapy containing berberine, amoxicillin and rabeprazole is noninferior to bismuth‐containing quadruple therapy in the initial treatment for *H. pylori* eradication.

## Graphical abstract

We demonstrated in a prospective randomized controlled trial that triple therapy containing berberine, amoxicillin, and rabeprazole can be used as the first‐line treatment for patients with primary *Helicobacter pylori* (*H. pylori*) infection. Abbreviation: UBT, urea breath test

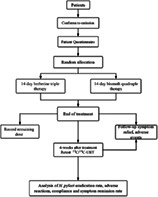

## 1 INTRODUCTION

*Helicobacter pylori* (*H. pylori*) infection can be detected in half the world population and 42.01%–84.62% of the population in China.^1^ Studies have revealed that *H. pylori* is a nonnegligible pathogenic factor in various gastrointestinal (GI) diseases such as peptic ulcer, gastric cancer (GC), and gastric mucosa‐associated lymphoid tissue (MALT) lymphoma. In addition, *H. pylori* infection is related to extra‐GI diseases including diabetes mellitus, coronary heart disease, and hypertension, etc.^2^, ^3^, ^4^ Besides its effectiveness on alleviating GI symptoms and ulcer healing, *H. pylori* eradication is also important for a complete remission of low‐grade gastric MALT lymphoma at an early stage.^5^, ^6^, ^7^, ^8^ In 2015, the Kyoto consensus defined *H. pylori* gastritis as an infectious disease, and recommended eradication therapy for all adult patients infected with *H. pylori*. Theoretically, the eradication rate of should reach 80% or higher, and that of the optimal treatment scheme should be over 95%.^9^ However, the validity of *H. pylori* eradication therapy shows a gradual downward trend globally. Eradication failure is mainly attributed to antibiotic resistance, variation among bacterial strains and hosts, as well as ineffective treatments.

Due to the increasing antibiotic resistance, it is difficult to achieve an eradication rate of 80% by using triple therapy.^10^ Bismuth‐containing quadruple therapy is therefore recommended as a well‐established first‐line empirical treatment regimen for *H. pylori* eradication.^11^ The resistance rates to amoxicillin, tetracycline, and furazolidone remain low (≤5%), while the resistance/multiresistance rate to other recommended antibiotics is high. Studies have revealed that an increased rate of dual metronidazole and clarithromycin resistance is one of the main causes of treatment failure.^12^

Berberine, a traditional Chinese medicine, has long been used in clinical practice as an antibacterial and anti‐inflammatory drug in China, which also has a good inhibitory effect on multidrug‐resistant *H. pylori* strains.^13^, ^14^ Previous studies have confirmed that treatment regimen including berberine can not only eradicate *H. pylori* effectively, but decrease the adverse events (AEs) to a certain extent, leading to a good patient compliance.^15^, ^16^, ^17^ Our previous studies have shown that quadruple therapy containing bismuth, berberine hydrochloride and amoxicillin achieves satisfactory eradication effect as both primary and rescue eradication therapy, which is comparable to the conventional bismuth‐containing quadruple regimen as recommended by consensus.^15^, ^17^ AEs of *H. pylori* eradication therapy were reported to be mainly linked to the use of antibiotics and bismuth. Bismuth can promote the efficacy of *H. pylori* eradication by protecting the mucosal tissue against damage and increase *H. pylori* susceptibility to antibiotics.^18^, ^19^ Nevertheless, the eradication rate using *H. pylori*‐sensitive antibiotics cannot be generally enhanced by the addition of bismuth.^20^

Considering a high susceptibility to amoxicillin and almost no resistance to berberine hydrochloride, we speculate that in the bismuth‐based quadruple regimen containing berberine hydrochloride and amoxicillin, bismuth may not improve the susceptibility to amoxicillin and berberine hydrochloride. The triple regimen including berberine hydrochloride combined with amoxicillin and a proton pump inhibitor (PPI) may obtain an equivalent effectiveness to that of the bismuth‐containing quadruple therapy in *H. pylori* eradication. In this this single‐center, randomized controlled, noninferiority study, we aimed to discriminate the effectiveness and safety of the triple regimen composed of berberine hydrochloride, PPI, and amoxicillin as the first‐line eradication therapy for *H. pylori* infection.

## 2 PATIENTS AND METHODS

This open‐label, randomized controlled, prospective, noninferiority study was conducted at the Xijing Hospital (Xi'an, Shaanxi Province, China) from January 2021 to July 2021. The study was approved by the Ethics Committee of Xijing Hospital (no. KY20202119) and followed the CONSORT Statement of Randomized Controlled Studies and the Declaration of Helsinki (Brazil, 2013). Written informed consent was obtained from each patient before their enrollment. The study was registered on ClinicalTrials.gov (registration no. NCT04697186).

### 2.1 Patient enrollment

Subjects aged 18–70 years who were confirmed to have initial *H. pylori* infection, dyspepsia, and had never received eradication therapy were recruited in this study. Exclusion criteria were: (a) hypersensitivity to study drug; (b) administration of antibiotics within 1 month or thrice a week and/or PPI within 2 weeks before ^13^C‐urea breath test (UBT); (c) with severe comorbidities; (d) with dysphagia, gastroduodenal ulcer, MALT lymphoma, or malignancy; (e) with pathologically confirmed high‐grade intraepithelial neoplasia; (f) with coagulation dysfunction, evident bleeding or iron deficiency anemia; (g) sustained use of hormones or nonsteroidal, anti‐inflammatory drugs (NSAIDs), anticoagulants or platelet aggregation inhibitors (except aspirin use <100 mg/day); (h) previous history of drug or alcohol abuse; (i) pregnant or lactating; (j) with psychological disorders; (k) had participated in other clinical trials during the past 3 months before the enrollment; and (l) refused to sign informed consent.

The enrolled patients were then randomly allocated to the berberine triple scheme (the observation group) or bismuth‐containing quadruple treatment (the control group) at a ratio of 1:1. The berberine triple therapy group were treated for 14 days with berberine hydrochloride (Yunnan Mingjing Pharmaceutical, Kunming, Yunnan Province, China) 300 mg thrice daily, amoxicillin (Zhuhai United Laboratories, Zhuhai, Guangdong Province, China) 1 g twice daily, and rabeprazole (Eisai China, Suzhou, Jiangsu Province, China) 10 mg twice daily. While the standard bismuth‐containing quadruple therapy group were treated for 14 days with rabeprazole (Eisai China) 10 mg twice daily, amoxicillin (Zhuhai United Laboratories) 1 g twice daily, clarithromycin (Laiyang Jiangbo Pharmaceutical, Laiyang, Shandong Province, China) 500 mg twice daily, and colloidal bismuth tartrate (Shanxi Xinbaoyuan Pharmaceutical, Datong, Shanxi Province, China) 200 mg twice daily. Rabeprazole and colloidal bismuth tartrate were both taken 30 minutes before meals, whereas amoxicillin, clarithromycin and berberine hydrochloride were administrated 30 minutes after meals.

### 2.2 Diagnosis of *H. pylori* infection and eradication efficacy

*H. pylori* infection was confirmed when any one of the ^13^C/^14^C‐UBT, *H. pylori* stool antigen test (*H. pylori* SAT), and rapid urease test (RUT) were positive. Negative ^13^C/^14^C‐UBT at 4 weeks after the completion of *H. pylori* eradication therapy was regarded as a successful eradication.

### 2.3 Patient outcomes

The major patient outcome was *H. pylori* eradication rate at 4 weeks after the completion of 14‐day eradication treatment. Secondary outcomes included improvement of clinical symptoms, patient compliance, and AEs. The factors that affected *H. pylori* eradication rate were also investigated.

Clinical symptoms of the patients at admission, including nausea, abdominal pain, bloating, diarrhea, constipation, belching, reflux, heartburn, were recorded. The severity and frequency of the symptoms were scored from 0 to 3 (0, none; 1, mild; 2, moderate; and 3, severe). The total score for each symptom was calculated using the following formula: total score = frequency score × severity score.

All patients were followed up via telephone during the trial to improve treatment compliance and to record their clinical symptom scores and AEs. Symptom relief and a good patient compliance were defined as a 50% reduction in the total symptom score and at least 80% of medications were taken, respectively.

### 2.4 Calculation of sample size and statistical analysis

Based on 69%–91% eradication rates with the bismuth‐containing quadruple therapy, an 85% eradication rate was assumed to be achieved in the two groups.^21^, ^22^, ^23^ The noninferiority δ of 10%, double‐sided α level of 0.05, and β power of 90% were considered, together with a 20% withdrawal rate. At least 262 patients in each group were required.

Continuous data were expressed as mean ± standard deviation, and were evaluated using the Student's *t*‐test. While categorical variables were expressed as numbers or percentages or frequencies, and were evaluated using the Chi‐square or Fisher's exact test. To determine noninferiority in the per‐protocol (PP) population, 95% confidence interval (CI) of the rate difference (RD) between the two groups was estimated by Student's *t*‐test. Using the lower limit of the RD 95% CI over −10%, the berberine triple therapy was not inferior to bismuth‐containing quadruple therapy, the intention‐to‐treat (ITT) population was also defined as statistically significant (*P <* 0.05).

## 3 RESULTS

### 3.1 Baseline characteristics of the patients

**FIGURE 1:**
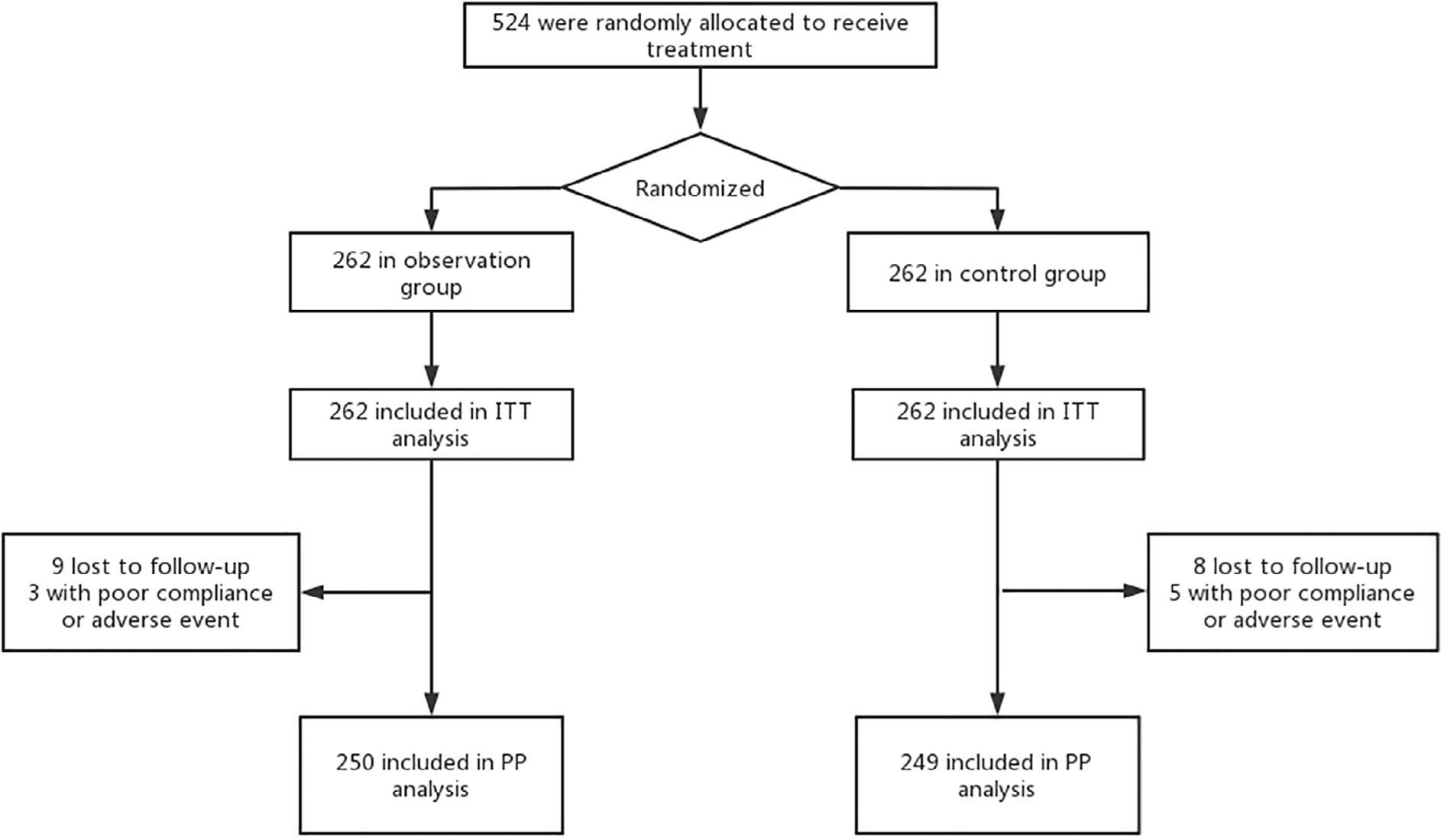
Flow diagram of the study. Abbreviations: ITT, intention‐to‐treat; PP, per‐protocol

From January 2021 to July 2021, 524 patients were successfully recruited in the trial, among whom 17 were lost to follow‐up, four discontinued against medical advice, one did not receive retesting at 4 weeks after the completion of *H. pylori* eradication therapy, and three withdrew from the trial due to severe allergic phenomena. Finally, 499 patients completed the full course of the trial (Figure 1 **)**. Baseline characteristics of the two groups are presented in Table 1.

**TABLE 1:** Baseline characteristics of the participants

Variables	Observation group (n = 262)	Control group (n = 262)	*P* value
Age, years (mean ± SD)	44.81 ± 12.08	45.73 ± 13.00	0.400
Sex, n (male/female)	137/125	128/134	0.432
BMI, kg/m^2^ (mean ± SD)	23.34 ± 3.29	22.86 ± 3.37	0.100
Ethnicity, n (%)			1.000
Han	256 (97.7)	256 (97.7)	
Others	6 (2.3)	6 (2.3)	
Educational level, n (%)			0.861
University and above	121 (46.2)	123 (46.9)	
High school and below	141 (53.8)	139 (53.1)	
Smoking, n (%)			0.419
Yes	63 (24.0)	51 (19.5)	
No	185 (70.6)	198 (75.6)	
Stop smoking	14 (5.3)	13 (5.0)	
Alcohol consumption, n (%)			0.833
Yes	61 (23.3)	57 (21.8)	
No	185 (70.6)	191 (72.9)	
Stop drinking	16 (6.1)	14 (5.3)	
Drinking water source, n (%)			0.287
Tap water	160 (61.1)	148 (56.5)	
Non‐tap water	102 (38.9)	114 (43.5)	
Decayed tooth, n (%)			0.544
Yes	68 (26.0)	62 (23.7)	
No	194 (74.0)	200 (76.3)	
History of drug allergy, n (%)			0.265
Yes	8 (3.1)	13 (5.0)	
No	254 (96.9)	249 (95.0)	
Long‐term medication, n (%)			0.415
Yes	41 (15.6)	48 (18.3)	
No	221 (84.4)	214 (81.7)	
Concomitant disease, n (%)			0.907
Yes	44 (16.8)	45 (17.2)	
No	218 (83.2)	217 (82.8)	
Family history, n (%)			0.612
Yes	62 (23.7)	67 (25.6)	
No	200 (76.3)	195 (74.4)	
Fruit intake, n (%)			0.730
5 units/week	61 (23.3)	65 (24.8)	
2–4 units/week	118 (45.0)	109 (41.6)	
1 unit/week	83 (31.7)	88 (33.6)	
Drinking tea, n (%)			0.448
6–7 days/week	49 (18.7)	53 (20.2)	
3–5 days/week	6 (2.3)	12 (4.6)	
1–2 days/week	41 (15.6)	43 (16.4)	
No	166 (63.4)	154 (58.8)	
Coffee drinking, n (%)			0.677
6–7 days/week	5 (1.9)	8 (3.0)	
3–5 days/week	4 (1.5)	2 (0.8)	
1–2 days/week	35 (13.4)	32 (12.2)	
No	218 (83.2)	220 (84.0)	
Dairy products, n (%)			0.857
5–7 times/week	52 (19.8)	55 (21.0)	
2–4 times/week	38 (14.5)	34 (13.0)	
Once weekly	172 (65.6)	173 (66.0)	
Vegetable intake, n (%)			0.465
≤250 g/day	54 (20.6)	43 (16.4)	
250–500 g/day	127 (48.5)	134 (51.1)	
>500 g/day	81 (30.9)	85 (32.4)	

Abbreviations: BMI, body mass index; SD, standard deviation.

### 3.2 *H. pylori* eradication rates

Of the 499 participants, only 78 experienced eradication failure. The eradication rate of the berberine triple therapy group was 79.8% (209/262) in the ITT analysis and 83.6% (209/250) in the PP analysis, while that of the standard bismuth‐containing quadruple therapy group were 80.9% (212/262) and 85.1% (212/249), respectively. No significant differences were observed between the two groups (ITT analysis, *P* =  0.742; PP analysis, *P*  =  0.636). Furthermore, the lower limit of RD 95% CI between the two groups was >−10% (−7.9% and −7.87%, respectively), indicating that the eradication rates were noninferior in both groups (Table 2).

**TABLE 2:** *Helicobacter pylori* eradication rates

	ITT analysis		PP analysis	
	Observation group	Control group	Observation group	Control group
n/N (%)	209/262 (79.8)	212/262 (80.9)	209/250 (83.6)	212/249 (85.1)
95% CI	74.4–84.5	75.6–85.5	78.4–88.0	80.1–89.3
RD 95% CI	−7.9, 5.7		−7.87, 4.87	
*P* value	0.742		0.636	

Abbreviations: CI, confidence interval; ITT, intention‐to treat; PP, per‐protocol; RD, rate difference.

### 3.3 Factors affecting *H. pylori* eradication rate

The factors affecting the validity of the two treatment strategies were analyzed in the PP population (Table 3), showing that the efficacy of the two treatment schemes was not affected by patients’ age, sex, body mass index (BMI), status of cigarette smoking, alcohol drinking, dietary habits, or AEs.

**TABLE 3:** Factors affecting the validity of *Helicobacter pylori* eradication in the per‐protocol population

Subgroup eradication rate, % (n/N)	Observation group (n = 250)	Control group (n = 249)
Age		
<43 years	87.2 (102/117)	89.4 (93/104)
≥43 years	80.5 (107/133)	82.1 (119/145)
*P* value	0.152	0.108
Sex		
Male	82.4 (108/131)	84.7 (105/124)
Female	84.9 (101/119)	85.6 (107/125)
*P* value	0.604	0.838
BMI		
<18.5 kg/m^2^	92.3 (12/13)	100 (25/25)
18.5–23.9 kg/m^2^	81.1 (107/132)	83.5 (116/139)
24.0–27.9 kg/m^2^	82.4 (70/85)	80.0 (52/65)
>28.0 kg/m^2^	100 (20/20)	95.0 (19/20)
*Z*	0.867	0.993
*P* value	0.386	0.321
Ethnicity		
Han	84.4 (206/244)	84.8 (207/244)
Others	50.0 (3/6)	100 (5/5)
*P* value	0.057	1.000
Education level		
University and above	86.2 (100/116)	88.9 (104/117)
High school and below	81.3 (109/134)	81.8 (108/132)
*P* value	0.300	0.117
Smoking		
Yes	84.5 (49/58)	91.8 (45/49)
No	84.3 (150/178)	83.0 (156/188)
Stop smoking	71.4 (10/14)	91.7 (11/12)
*P* value	0.448	0.242
Alcohol consumption		
Yes	89.7 (52/58)	81.5 (44/54)
No	82.4 (145/176)	85.6 (155/181)
Stop drinking	75.0 (12/16)	92.9 (13/14)
*P* value	0.272	0.531
Drinking water source		
Tap water	84.9 (129/152)	82.3 (116/141)
Non‐tap water	81.6 (80/98)	88.9 (96/108)
*P* value	0.500	0.146
Decayed tooth		
Yes	84.8 (56/66)	80.0 (48/60)
No	83.2 (153/184)	86.8 (164/189)
*P* value	0.750	0.199
History of drug allergy		
Yes	87.5 (7/8)	100 (13/13)
No	83.5 (202/242)	84.3 (199/236)
*P* value	1.000	0.226
Long‐term medication		
Yes	78.9 (30/38)	86.9 (40/46)
No	84.4 (179/212)	84.7 (172/203)
*P* value	0.400	0.741
Concomitant disease		
Yes	76.7 (33/43)	88.1 (37/42)
No	85.0 (176/207)	84.5 (175/207)
*P* value	0.182	0.555
Family history		
Yes	85 (51/60)	82.8 (53/64)
No	83.2 (158/190)	85.9 (159/185)
*P* value	0.737	0.544
Fruit intake		
5 units/week	84.5 (49/58)	85.9 (55/64)
2–4 units/week	84.1 (95/113)	82.5 (85/103)
1 unit/week	82.3 (65/79)	87.8 (72/82)
*Z*	0.366	0.421
*P* value	0.714	0.673
Drinking tea		
6–7 days/week	85.1 (40/47)	84.0 (42/50)
3–5 days/week	66.7 (4/6)	100 (12/12)
1–2 days/week	78.9 (30/38)	87.8 (36/41)
No	84.9 (135/159)	83.6 (122/146)
*Z*	0.531	0.642
*P* value	0.595	0.521
Coffee drinking		
6–7 days/week	100 (5/5)	85.7 (6/7)
3–5 days/week	100 (4/4)	100 (2/2)
1–2 days/week	80.0 (28/35)	90.6 (29/32)
No	83.5 (172/206)	84.1 (175/208)
*Z*	0.209	0.987
*P* value	0.835	0.324
Dairy products		
5–7 times/week	85.7 (42/49)	88.7 (47/53)
2–4 times/week	89.5 (34/38)	81.3 (26/32)
Once weekly	81.6 (133/163)	84.8 (139/164)
Z	1.046	0.418
*P* value	0.296	0.676
Vegetable intake		
<250 g/day	86.5 (45/52)	87.5 (35/40)
250–500 g/day	82.0 (100/122)	86.3 (107/124)
>500 g/day	84.2 (64/76)	82.4 (70/85)
*Z*	0.223	0.881
*P* value	0.823	0.378
Adverse events		
Yes	93.8 (15/16)	87.1 (27/31)
No	82.9 (194/234)	84.9 (185/218)
*P* value	0.433	0.954

Abbreviation: BMI, body mass index.

### 3.4 AEs, compliance, and symptom improvement

All patients received telephone follow‐up during the trial to improve treatment compliance and to record their clinical symptom scores and AEs. Compared with the standard bismuth‐containing quadruple therapy group, The overall rate of AEs in the berberine triple therapy group was relatively low (8.8% vs 16.0%, *P* = 0.012) (Table 4). Common AEs included nausea, diarrhea, dysgeusia, bloating, fever, anorexia, and weakness (excluding bismuth‐related melena), which improved spontaneously approximately 2 weeks after treatment was discontinued (Table 5). Compared to the standard bismuth‐containing quadruple therapy group, the incidence of dysgeusia was significantly lower in the berberine triple therapy group (4.3% vs 26.2%, *P* = 0.043). Differences were not observed in patient compliance and symptom relief between the two groups.

**TABLE 4:** Rates of adverse events, compliance, and symptom improvement

Variables (n/N, %)	Observation group	Control group	*P* value
Overall adverse events	23/262 (8.8)	42/262 (16.0)	0.012
Compliance rate	246/262 (93.9)	237/262 (90.5)	0.143
2‐week symptom improvement	207/250 (82.8)	207/249 (83.1)	0.921
6‐week symptom improvement	236/250 (94.4)	237/249 (95.2)	0.695

**TABLE 5:** Frequency of adverse events in the two groups

Adverse events (n, %)	Observation group (n = 23)	Control group (n = 42)	*P* value
Diarrhea	8 (34.8)	9 (21.4)	0.241
Nausea	11 (47.8)	11 (26.2)	0.078
Abdominal pain	2 (8.7)	4 (9.5)	1.000
Dizziness	1 (4.3)	3 (7.1)	1.000
Bloating	1 (4.3)	2 (4.8)	1.000
Dysgeusia	1 (4.3)	11 (26.2)	0.043
Anorexia	2 (8.7)	0 (0)	0.122
Rash	3 (13.0)	6 (14.3)	1.000
Vomiting	0 (0)	4 (9.5)	0.323
Fever	0 (0)	1 (2.4)	1.000
Fatigue	0 (0)	2 (4.8)	0.536

## 4 DISCUSSION

*H. pylori* is an infectious bacterium with a high global infection rate. It is closely related to both GI and extra‐GI diseases.^3^, ^4^, ^24^ In the whole‐age stage, regardless of whether the infected person has accompanying symptoms or not, *H. pylori* eradication may prevent the occurrence of GC.^25^, ^26^, ^27^ If *H. pylori* cannot be eradicated, the infection will continue for life.^28^ Current consensus recommends that eradication therapy should be implemented as long as *H. pylori* infection is detected, unless there are competing considerations, the initial therapy should achieve an ideal eradication success.^5^, ^11^ Successful initial eradication can avoid subsequent repeated treatment and testing, reducing medical costs, self‐anxiety, and negative effects on gut microbiota.^29^

Recently, the easy availability and extensive application of antibiotics have become the direct factors for mutation, secondary, and multidrug resistance of *H. pylori* strains. As the prevalence of antibiotic resistance increases, treatment regimens for *H. pylori* eradication continue to escalate from the initial dual regimen (PPI plus amoxicillin), a 7‐day triple regimen (PPI plus two antibiotics), to the 14‐day bismuth‐containing quadruple regimen. An eradication rate below 80% using the triple therapy is no longer acceptable.^10^ In China, the resistance rate of *H. pylori* to clarithromycin (20%–55.2%), metronidazole (40%–90.6%), and levofloxacin (20%–50%) is high, while that to amoxicillin (0%–7.7%), tetracycline (0%–5%), and furazolidone (0%–0.91%) remains low.^30^, ^31^, ^32^ However, it is difficult to exert too much effect clinically due to numerous AEs, complicated administration, and nonavailability. Therefore, the selection of treatment strategies recommended by the guidelines remain limited and may not reach a satisfactory level.^33^, ^34^

High‐dose dual therapy with PPI and amoxicillin has been recommended as both initial and rescue therapies for *H. pylori* eradication. Increasing the drug dose without changing the dosing frequency of PPI–amoxicillin dual therapy has comparable efficacy and patient compliance for *H. pylori* eradication compared with conventional mainstream guidelines.^35^, ^36^ While changing the frequency of drug regimen without changing the dose, PPI–amoxicillin dual therapy administered four times daily achieved better efficacy and safety than currently recommended regimens (especially first‐line therapy), mainly in Asia.^37^ However, despite the increasing eradication rate, a decreasing patient compliance to a frequent drug dosing and a possible increase of AEs related to large‐dose drug use may influence the efficacy and safety of the treatment regimes. Therefore, more studies are needed to further improve the efficacy of first‐line eradication therapy of *H. pylori*.

Berberine is an alkaloid derived from *Coptis chinensis* and possesses various biological activities. It is widely used in clinical practice because of the unique antimicrobial, antibacterial, and anti‐inflammatory effects. The potency of berberine on reducing the activity of *H. pylori* is seldom affected by the internal and external environments, especially against multidrug‐resistant strains. This effect is significantly enhanced with clarithromycin, which may be due to a synergy between them.^38^, ^39^, ^40^ Berberine could specifically inactivate urease by combining with the sulfhydryl group of urease active site (nickel metal center) in the dose‐dependent manner. Furthermore, berberine promotes the separation of nickel from the urease dimer and interfered with the maturation of urease.^41^, ^42^

Clinical studies have found that berberine combined with traditional triple therapy or alternatives to antibiotics with high resistance rate in bismuth‐containing quadruple regimen shows higher *H. pylori* eradication and cure rates in the treatment of *H. pylori*‐related gastritis and peptic ulcer. It also reduces the recurrence rate of ulcers.^15^, ^16^, ^17^ In addition, berberine interacts with antibiotics, illustrating that it has a certain eradication consequence on multidrug‐resistant *H. pylori* strains when used in combination with other antibiotics.^14^

The *H. pylori* nomogram model has demonstrated that bismuth can enhance the eradication rate by at least 30%,^18^, ^19^ though it is unlikely to improve the treatment effectiveness.^20^ In addition, compared with PPIs, bismuth may cause a higher rate of AEs.

The results of our study showed that eradication rate of *H. pylori* in the PP analysis was above 80%, and there was no statistical difference between the berberine triple therapy and the bismuth‐containing quadruple therapy. Furthermore, the lower limit of the RD 95% CI between the two groups was >−10% (−7.9% and −7.87%, respectively), indicating that the eradication rate was noninferior in both the berberine triple therapy group and the standard bismuth‐containing quadruple therapy group. In addition, clinical symptom improvement and patient compliance were comparable between the two groups. However, the overall incidence of AEs in the berberine triple therapy group was relatively low (8.8% vs 16.0%, *P* = 0.012). Among all AEs, compared to the standard bismuth‐containing quadruple therapy group, dysgeusia rarely occurred in the berberine triple therapy group (4.3% vs 26.2%, *P* = 0.043). We speculate that this may be related to the combined use of bismuth and clarithromycin in the quadruple therapy. AEs in both groups were mild or moderate, which disappeared about 2 weeks after treatment was discontinued. Considering its eradication effectiveness, good safety, low medical cost and easy availability, we suggest a combined use of berberine hydrochloride, amoxicillin, and rabeprazole as a new triple regimen with excellent clinical prospects.

The study of Shang et al^43^ indicated that the resistance rate of *H. pylori* to clarithromycin in Gansu Province, China was 54.72%, and the dual, triple, and quadruple resistance rate exceeded 20%. Xie et al yielded similar results that exceeded 30% in northwest China.^44^ According to the Chinese consensus, bismuth‐containing quadruple treatment is the preferred regimen in northwest China. We did not use berberine, amoxicillin, bismuth, and rabeprazole as control in the current study, because our previous study has compared efficacy of a modified quadruple therapy of PPI, berberine, amoxicillin and bismuth with that of the standard bismuth‐containing quadruple therapy, showing a comparable eradication rate.^45^ It is speculated that the effectiveness of berberine, amoxicillin, and rabeprazole triple therapy and berberine, amoxicillin, bismuth quadruple therapy is equivalent. This also confirms that *H. pylori* is not resistant to berberine. As drug resistance rate to amoxicillin is low, the combination of berberine and amoxicillin is *H. pylori*‐sensitive.

Our subgroup analysis of the factors affecting the eradication rate showed that the efficacy of both regimens was not affected by patient's age, sex, BMI, smoking, alcohol consumption, dietary habits, or AEs. It might be due to the small sample size of this study. Further confirmation of our results with studies with large sample sizes is needed.

There were some limitations to our study. First, the vast majority of subjects in the study were from northwest China, and further studies are required to verify whether the berberine triple regimen is feasible in other regions. Second, quadruple therapy of berberine, amoxicillin, rabeprazole, and bismuth was not used as an additional control, and the effect of bismuth could not have been fully excluded. Finally, gastric pH value, cytochrome P450 2C19 (CYP2C19) genotype, and antibiotic susceptibility of *H. pylori* in our patients were not assessed.

In conclusion, our study preliminarily demonstrated the efficiency and safety of the new triple regimen including berberine hydrochloride, amoxicillin, and rabeprazole for initial eradication of *H. pylori*, providing an innovative eradication regimen of *H. pylori*. Furthermore, 2‐week berberine triple regimen was well tolerated with an acceptable eradication rate and low AEs. This triple therapy can be considered for *H. pylori* eradication in the local region.

## CONFLICT OF INTEREST

The authors declared that they had no conflicts of interest.
